# Connectivity-based fixel enhancement: Whole-brain statistical analysis of diffusion MRI measures in the presence of crossing fibres

**DOI:** 10.1016/j.neuroimage.2015.05.039

**Published:** 2015-08-15

**Authors:** David A. Raffelt, Robert E. Smith, Gerard R. Ridgway, J-Donald Tournier, David N. Vaughan, Stephen Rose, Robert Henderson, Alan Connelly

**Affiliations:** a Florey Institute of Neuroscience and Mental Health, Melbourne, Victoria, Australia; b FMRIB Centre, Nuffield Department of Clinical Neurosciences, University of Oxford, Oxford, UK; c Wellcome Trust Centre for Neuroimaging, UCL Institute of Neurology, London, UK; d Department of Biomedical Engineering, Division of Imaging Sciences and Biomedical Engineering, King's College London, London, UK.; e Centre for the Developing Brain, King's College London, London, United Kingdom; f Florey Department of Neuroscience and Mental Health, University of Melbourne, Melbourne, Victoria, Australia.; g Department of Medicine, Austin Health and Northern Health, University of Melbourne, Melbourne, Victoria, Australia.; h The Australian e-Health Research Centre, CSIRO-Digital Productivity Flagship, Royal Brisbane and Women's Hospital, Herston, Australia; i Department of Neurology, Royal Brisbane and Women's Hospital, Herston, Australia

**Keywords:** AFD, apparent fibre density, AFROC, alternative free-response receiver operator curve, AUC, area under the curve, CFE, connectivity-based fixel enhancement, CHARMED, composite hindered and restricted model of diffusion, CUSP-MFM, cube and sphere multi-fascicle model, DWI, diffusion-weighted imaging, FA, fractional anisotropy, Fixel, a specific fibre population within a voxel, FBA, fixel-based analysis, FOD, fibre orientation distribution, FWE, family-wise error, FWHM, full width at half maximum, HMOA, hindrance modulated orientational anisotropy, MD, mean diffusivity, MND, motor neurone disease, MRI, magnetic resonance imaging, ROC, receiver operator curve, ROI, region of interest, SIFT, spherical deconvolution informed filtering of tractograms, SNR, signal to noise, SPM, statistical parametric mapping, TBSS, tract-based spatial statistics, TFCE, threshold-free cluster enhancement, VBA, voxel-based analysis, Diffusion, MRI, Statistics, Fixel, Connectivity, Analysis

## Abstract

In brain regions containing crossing fibre bundles, voxel-average diffusion MRI measures such as fractional anisotropy (FA) are difficult to interpret, and lack within-voxel single fibre population specificity. Recent work has focused on the development of more interpretable quantitative measures that can be associated with a specific fibre population within a voxel containing crossing fibres (herein we use *fixel* to refer to a specific *fibre population* within a single *voxel*). Unfortunately, traditional 3D methods for smoothing and cluster-based statistical inference cannot be used for voxel-based analysis of these measures, since the local neighbourhood for smoothing and cluster formation can be ambiguous when adjacent voxels may have different numbers of fixels, or ill-defined when they belong to different tracts. Here we introduce a novel statistical method to perform whole-brain fixel-based analysis called connectivity-based fixel enhancement (CFE). CFE uses probabilistic tractography to identify structurally connected fixels that are likely to share underlying anatomy and pathology. Probabilistic connectivity information is then used for tract-specific smoothing (prior to the statistical analysis) and enhancement of the statistical map (using a threshold-free cluster enhancement-like approach). To investigate the characteristics of the CFE method, we assessed sensitivity and specificity using a large number of combinations of CFE enhancement parameters and smoothing extents, using simulated pathology generated with a range of test-statistic signal-to-noise ratios in five different white matter regions (chosen to cover a broad range of fibre bundle features). The results suggest that CFE input parameters are relatively insensitive to the characteristics of the simulated pathology. We therefore recommend a single set of CFE parameters that should give near optimal results in future studies where the group effect is unknown. We then demonstrate the proposed method by comparing apparent fibre density between motor neurone disease (MND) patients with control subjects. The MND results illustrate the benefit of fixel-specific statistical inference in white matter regions that contain crossing fibres.

## Highlights

• We introduce the fixel—a specific fibre population within a voxel.
• A novel method for whole-brain fixel-based analysis of diffusion MRI is presented.
• Structural connectivity between fixels is derived from template-based tractography.
• Connectivity information is used for tract-specific smoothing and enhancement.
• Quantitative assessment and an *in vivo* demonstration is performed.

## Graphical abstract

**Figure:**
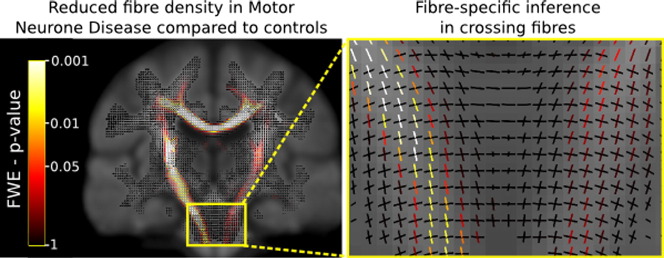

## Introduction

Voxel-based analysis (VBA) is an image analysis technique for performing whole-brain voxel-wise statistical tests across and within groups of subjects, originally introduced in the form of statistical parametric mapping (SPM; Friston et al., 1991). A particular strength of the VBA approach is that, in addition to enabling specific hypotheses to be tested, it has the ability to localise group differences or correlations without any prior spatial hypothesis. Over the last two decades, VBA has been applied in many fields of neuroimaging to investigate quantitative information derived from image intensity (e.g. positron emission tomography (Worsley et al., 1992) and functional MRI (Friston et al., 1995)) and image morphology (e.g. voxel-based morphometry (Ashburner and Friston, 2000) and tensor-based morphometry (Ashburner, 2000; Gee, 1999)).

VBA commonly involves four key steps:

1. Obtain anatomical correspondence by transforming all subject images to a common template using an image registration algorithm.
2. Smooth images to boost the signal-to-noise ratio, alleviate registration misalignments, and improve the normality of residuals when performing parametric statistical analysis.
3. Perform a statistical test at each voxel resulting in a test-statistic image (also known as a statistical parametric map).
4. Statistical inference (assign p-values to voxels, peaks or clusters of voxels).

One caveat in VBA is the need to account for the large number of multiple tests during statistical inference. Random field theory (RFT) (Worsley et al., 1992) and non-parametric permutation testing (Nichols and Holmes, 2002) are two commonly used methods to compute family-wise error (FWE) corrected p-values. While these methods can be used to make voxel-level inferences, they can also be applied to derive FWE-corrected p-values for clusters of contiguous voxels above a predefined threshold (Friston et al., 1994; Poline and Mazoyer, 1993). Cluster-level inference can be more sensitive than voxel-level inference by exploiting spatial correlations in voxel intensities due to shared underlying anatomy and pathology (Friston et al., 1996).

In the field of diffusion-weighted imaging (DWI), VBA is being used increasingly to study white matter development, aging and pathology. The vast majority of these studies have involved quantitative measures derived from the diffusion tensor model, such as mean diffusivity (MD) and fractional anisotropy (FA) (Basser and Pierpaoli, 1996). Since these tensor-derived measures are scalar quantities, traditional VBA software packages (such as SPM (www.fil.ion.ucl.ac.uk/spm/) and FSL (www.fmrib.ox.ac.uk/fsl)) can be used to analyse the resultant 3D images. More recently, several diffusion-specific VBA approaches have been proposed that perform statistics on a tract skeleton (Smith et al., 2006) or surface (Maddah et al., 2011; Yushkevich et al., 2008; Zhang et al., 2010). By projecting local quantitative measures onto a tract skeleton or 2D surface, these methods aim to reduce the impact of imperfect image registration on anatomical correspondence. However, not all white matter tracts can be modelled by a skeleton or surface, and therefore these methods suffer from other problems related to inaccurate tract representation and projection (Bach et al., 2014).

**Fig. 1:**
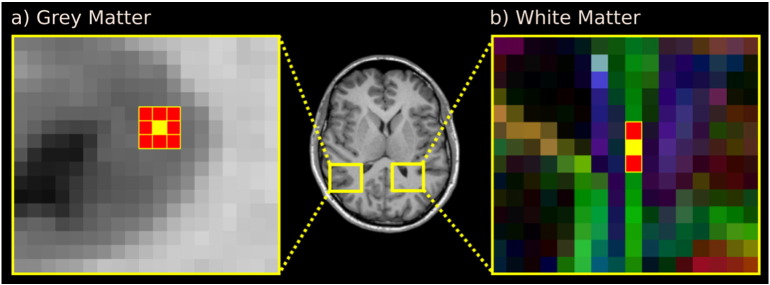
a) In grey matter, it is reasonable to assume image intensities are spatially correlated with neighbours isotropically for the purposes of smoothing and cluster formation. Illustrated in yellow is a voxel of interest with neighbouring voxels coloured red. b) White matter anatomy is oriented and extended in nature, therefore an isotopic neighbourhood is not appropriate. Shown is a fractional anisotropy map coloured by the direction of the primary tensor eigenvector (red: left–right, green: anterior–posterior, blue: inferior–superior). Not all voxels adjacent to the voxel of interest (yellow voxel within the optic radiation) are relevant for smoothing and cluster formation since neighbouring voxels contain different fibre tracts (e.g. tapetum of corpus callosum and arcuate fasciculus). In this example only the voxels anterior and posterior (shown in red) should be considered as neighbours for clustering and smoothing.

Two issues relevant to VBA of white matter that have been largely neglected to date are as follows:

1. A white matter voxel can contain multiple populations of fibres, each belonging to a specific white matter tract with a unique function (a scenario often referred to as crossing fibres). Recent evidence suggests up to 90% of white matter voxels contain two or more fibre populations (Jeurissen et al., 2012). Ideally VBA of diffusion MRI should be able to attribute any significant effect to a specific fibre population in regions with crossing fibres.
2. White matter contains anatomical structures that are oriented and can span many voxels in the image. Spatially distant voxels can share the same underlying anatomy, yet adjacent voxels may share no anatomy (e.g. at a bundle interface). It is therefore reasonable to assume that correlations in quantitative measures can occur anywhere *along* a fibre tract, but not necessarily with all voxel neighbours isotropically (as is assumed to be the case in grey matter) (see Fig. 1).^1^ This is based on the assumption that axons are likely to be affected by development, pathology or aging along their entire length.

Both issues 1 and 2 are problematic for appropriate smoothing and cluster-based statistical inference. A neighbourhood for traditional isotropic smoothing and cluster formation is ambiguous when adjacent voxels have multiple fibre populations, and ill-defined when adjacent fibre populations belong to different fibre tracts. Note that in the aforementioned surface- and tract-skeleton-based methods (Maddah et al., 2011; Smith et al., 2006; Yushkevich et al., 2008; Zhang et al., 2010), parameterisation of the tract enables smoothing and clustering with a more appropriate neighbourhood. However, 2D surfaces or 3D skeletons cannot appropriately represent all white matter tracts (e.g. fanning of the corpus callosum), and current methods do not account for crossing fibres.

In recent years, a number of quantitative measures have been proposed that can be assigned to a specific fibre population within a given voxel. Here we coin the term *fixel*^2^ to refer to a specific *population of fibres* within a single *voxel*. We note that while voxel-average summary statistics such as generalized fractional anisotropy (Tuch, 2004) can be used to characterise model-free data derived via Diffusion Spectrum Imaging (Wedeen et al., 2005) or Q-Ball imaging (Tuch, 2004), it is impossible to derive fixel-specific quantitative measures without making some assumptions about the diffusion within a single fixel. As a consequence, all fixel-specific quantitative measures to date are based on mixture models. For example, in the ‘composite hindered and restricted model of diffusion’ (CHARMED) model, the volume fraction of each fixel is estimated by assuming a restricted model of diffusion for each fixel (Assaf and Basser, 2005). In a similar concept, the apparent fibre density (AFD) and hindrance modulated orientational anisotropy (HMOA) are measures also related to the volume of the intra-axonal restricted compartment (Dell'Acqua et al., 2013; Raffelt et al., 2012b). The motivation behind these measures is that the intra-axonal restricted compartment should be sensitive to various white matter pathologies that affect the number of axons. In more recent work, Scherrer and Warfield (2012) use a novel acquisition scheme and fitting procedure (called cube and sphere multi-fascicle model, CUSP-MFM) to model the intra-axonal diffusion for each fixel with a diffusion tensor. In CUSP-MFM fixel-specific volume fractions and diffusivities can be estimated.

All of these fixel-based measures have the potential to give more specific information than tensor-derived measures by identifying specific white matter tracts that are affected in regions with crossing fibres. However, due to issues 1 and 2 outlined above, traditional 3D statistical software packages cannot be applied to perform VBA on these fibre-specific measures.

In this work we propose a novel statistical framework entitled connectivity-based fixel enhancement (CFE) for performing group comparisons or correlations of fixel-specific measures within all of the white matter (i.e. a fixel-based analysis, FBA). We use whole-brain probabilistic tractography on a group average template to define the connectivity between each fixel and all other fixels in the brain, and use this fixel-fixel connectivity information for both smoothing (i.e. fixel-specific measures are smoothed only with other fixels that share common streamlines), and to boost the belief in (enhance) the test-statistic of each fixel based on information from structurally connected fixels. We investigate the proposed method using quantitative simulations, and demonstrate its utility by comparing a cohort of motor neurone disease patients with healthy controls.

## Methods

### Fixel–fixel connectivity

**Fig. 2:**
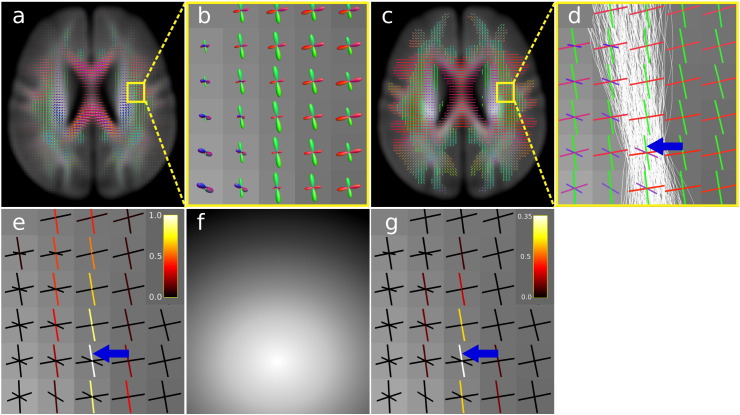
Illustration of fixel–fixel connectivity and smoothing. a) A group-average FOD template colour-coded by direction (red: left–right, blue: inferior–superior, green: anterior–posterior). b) Zoomed in region from a, showing individual FODs within the group-average FOD template. c) The direction and number of fixels in each voxel was computed by segmenting each FOD in a (coloured by fixel orientation). d) A single exemplar fixel *f* (blue arrow, belonging to the superior longitudinal fasciculus), with associated probabilistic streamlines. e) Fixels colour-coded by ‘connectivity’ to fixel *f*. The connectivity, *c*_*fi*,_ between exemplar fixel *f* and fixel *i* is defined as the proportion of streamlines traversing fixel *f* that also traverse fixel *i*. f) A spatial Gaussian kernel centred on fixel, *f*, is multiplied with fixel connectivity in d to estimate the fixel-specific smoothing neighbourhood weights shown in f. g) Smoothing neighbourhood weights for fixel, *f*, used to smooth fixel data prior to analysis.

To visually demonstrate the concept of fixel connectivity, consider the example shown in Fig. 2. Fig. 2a, b shows a group-average template generated via registration of fibre orientation distribution (FOD) images (Raffelt et al., 2011). The location and direction of all white matter fixels can be computed via segmentation of each FOD lobe in the group-average template (Fig. 2c). FOD segmentation was performed using the method outlined in Smith et al. (2013), which involves segmenting each lobe/fibre using zero crossings of the FOD and their directions based on peak amplitude (note that while we use the spherical deconvolution model in this example, some diffusion MRI models compute fixels directly, and therefore may not require an explicit segmentation step). For this example, consider fixel *f* indicated by the blue arrow in Fig. 2d. Probabilistic streamlines are used to compute the connectivity to all other white matter fixels (Fig. 2d shows only those streamlines extracted from the whole-brain tractogram that traverse fixel *f*). We define the connectivity from fixel *f* to fixel *i*, *c_fi_*, as the proportion of the streamlines traversing fixel *f* that also traverse fixel *i*. Note that since *c_fi_* is the number of shared streamlines relative to all streamlines associated with *f*, this measure is not symmetric, (i.e. *c_fi_* ≠ *c_if_*). In Fig. 2e, each fixel is coloured by *c_fi_*: this demonstrates how the use of probabilistic tractography provides a mechanism to quantify fixel–fixel connectivity based on uncertainty in the estimated fibre orientations, i.e. we are more confident that fixels with a high density of the dispersing streamlines are likely to share underlying anatomy (and therefore be correlated) with fixel *f*.

When computing the whole-brain fixel–fixel connectivity matrix, streamlines are assigned to fixels in the template based on the local streamline tangent. The streamline tangent is computed by the entry and exit point through the voxel. For practical reasons, we remove all fixel–fixel connectivity values, *c*_i,_ that are less than 0.01. This eliminates many fixels connected by spurious probabilistically-unlikely streamlines, and also increases the sparsity (and therefore decreases the required memory) of the whole-brain fixel–fixel connectivity matrix.

### Connectivity-based smoothing

The first application of fixel–fixel connectivity is to weight neighbourhood fixels for the purposes of pre-smoothing data. In 3D voxel-based analysis data is typically smoothed with a local isotropic neighbourhood using a Gaussian kernel. In this work we also smooth locally, however we compute smoothing weights (Fig. 2g) by multiplying Gaussian kernel weights (Fig. 2f) with the fixel–fixel connectivity weights (Fig. 2e). Connectivity-based smoothing ensures that fixel-specific measures are smoothed locally with fixels belonging to the same fibre tract, and preferentially smooths data with fixels with high connectivity values, whose fixel data are most likely to correlate strongly with that of the fixel of interest. We note that smoothing could be achieved by using connectivity weights only (Fig. 2e), since values are larger in local fixels due to probabilistic streamline dispersal. However, by spatially restricting smoothing with a Gaussian kernel, data are less likely to be smoothed with remote fixels containing very different values. This may be an issue for some quantitative measures that vary along a bundle’s length (e.g measures related to fixel volume fraction will vary based amount of crossing with other fibres).

### Connectivity-based fixel enhancement

The second application of fixel–fixel connectivity is in statistical inference. Here we present a novel approach for fixel-based statistics called connectivity-based fixel enhancement (CFE).

Conventional cluster-based statistical analysis involves applying a pre-specified threshold to the test-statistic image to identify co-located (clustered) voxels. The motivation behind cluster-based analysis is to identify extended areas of group differences that are more spatially extended than would be expected due to the noise coherence alone. Once clusters of voxels have been identified, the likelihood (p-value) that each cluster (of a certain size) has occurred due to chance can be computed by comparing the cluster size to the null distribution of cluster sizes (estimated via Gaussian random field theory (Worsley et al., 1992) or permutation testing (Holmes et al., 1996)).

One dilemma in any method for cluster-based inference is the choice of an arbitrary threshold. While the choice of threshold does not impact on the validity of the results, it can greatly affect the outcome and therefore complicate scientific interpretation. Smith and Nichols (2009) proposed an alternative to threshold-based cluster analysis called “threshold-free cluster enhancement” (TFCE). In the Smith and Nichols (2009) 3D TFCE implementation, the enhanced test-statistic at voxel *v*, is equal to the sum of the cluster extents, *e*, as the statistic image is thresholded at various heights, *h*, up to the height of *v*, *h_v_*. More specifically TFCE is defined as:

(1) $$\mathrm{TFCE}\left(v\right)=\int_{0}^{hv}e{\left(h\right)}^{E}h^{H}dh$$

with default values of constants *E* = 0.5, *H* = 2. Note that these defaults are justified by theory and empirical results in Smith et al. (9). By setting *H* to more than 1 the TFCE output gives more weight to extents (clusters) at larger levels of *h*, while setting *E* to less than 1 ensures the TFCE output scales less than linearly with cluster size (something that is desirable at low thresholds when clusters are large and do not provide useful spatial specificity (Smith and Nichols, 2009)).

CFE is a TFCE-like approach that exploits connectivity information to enhance the test-statistic of each fixel based on the support lent to it by other structurally connected fixels. In the original TFCE paper (Smith and Nichols, 2009), the cluster extent *e* is defined as the number of supra-threshold voxels spatially connected to voxel *v*. However in CFE, we redefine *e* as the weighted sum of fixels structurally connected to the fixel being enhanced, *f*. Precisely, CFE is defined as:

(2) $$CFE\left(f\right)=\int_{0}^{hf}e{\left(f,h\right)}^{E}h^{H}dh$$

(3) $$e\left(f,h\right)=\sum_{i=1}^{n\left(h\right)}{c_{fi}}^{C}$$

where *n*(*h*) is the total number of supra-threshold fixels connected to *f*, *c*_fi_ is the connectivity defined as the proportion of streamlines traversing fixel *f* that also traverse fixel *i*, and *C* is a constant. By weighting each fixel by *c_fi_*, highly connected fixels (i.e. those that we are more certain share many axons) contribute more to the enhancement than fixels with low connectivity. Furthermore, *c_fi_* is raised to the power *C*, which enables the option to modulate the strength of this connectivity dependent enhancement. For example when *C* = 0 all connected fixels contribute evenly to the enhancement, whereas when *C* = 1 they contribute with a weight proportional to their measured connectivity.

It is worth emphasising that in the original TFCE method, a voxel may contribute to the enhancement of another only if they are *spatially connected* by coexisting within a supra-threshold cluster. However in CFE, a supra-threshold fixel may enhance another as long as it is *structurally connected*, without any requirement that the fixels are spatially connected within a suprathreshold cluster. This distinction arises from the fact that in CFE, we have additional information provided by tractography. This enables us to determine whether fixels are likely to share underlying anatomy and pathology, without any need to assume that supra-threshold fixels must be spatially contiguous.

### Illustrative example

**Fig. 3:**
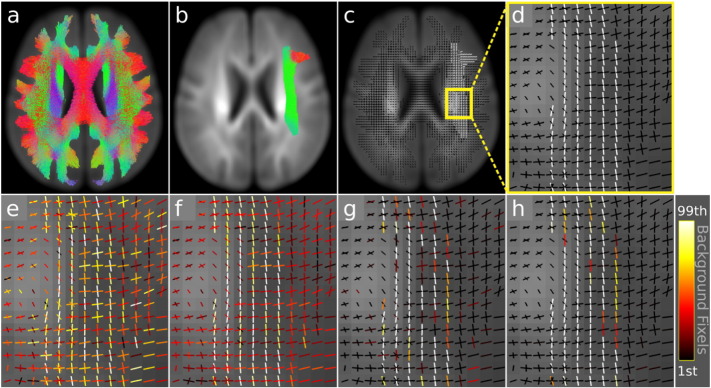
Illustrative example. a) Whole-brain probabilistic tractogram computed on the group-average FOD template. Streamlines were used to derive fixel–fixel connectivity for smoothing and enhancement. Streamlines are coloured by direction (red: left–right, blue: inferior–superior, green: anterior–posterior). b) A tract-of-interest, the arcuate fasciculus, was extracted from the whole-brain tractogram in a (note that only streamlines belonging to the slice are shown). c) Individual fixels belonging to the arcuate fasciculus were identified based on streamline visitation. All arcuate fixels were assigned a ‘signal’ of one. d) Zoomed in region of the ‘signal only’ image in c showing the arcuate fasciculus fixels in white and background (zero) fixels in black. e) Signal + noise image after adding Gaussian noise (signal-to-noise of 2) to the signal only image in d. f) Connectivity-based smoothing of e. g) CFE of e. h) Both connectivity-based smoothing and CFE of e. To best visualise the separation of signal from background, all images e–f are windowed based on the 1st to 99th percentile of the background fixel values.

Fig. 3 contains an illustrative example of connectivity-based smoothing and CFE enhancement to an artificially generated signal + noise image. A tract-of-interest (the arcuate fasciculus, Fig. 3b) was extracted from the whole-brain tractogram (Fig. 3a) computed on the FOD template (Fig. 2a, b) (see the following section for details). Fixels belonging to the arcuate fasciculus were identified (via streamline visitations) and assigned a signal value of one (Fig. 3d). All non-arcuate (background) fixels were assigned a value of zero. A signal + noise image was created by adding random Gaussian noise with a standard deviation of 0.5, corresponding to a signal-to-noise ratio of 2 (Fig. 3e). We then applied the following enhancements to separate the signal from the noise:

• Connectivity-based smoothing only, FWHM = 10 mm (Fig. 3f);
• CFE only (no smoothing), *E* = 1, *H* = 2, *C* = 0.5 (Fig. 3g);
• Connectivity-based smoothing then CFE, FWHM = 10 m, *E* = 1, *H* = 2, C = 0.5 (Fig. 3h).

To best visualise that the arcuate ‘signal’ fixels are separated from the background fixels, all images in Fig. 3e–h are windowed such that the colour bar range extends from the 1st to 99th percentile of the background fixel values. Fixels indicated in white are therefore larger than (separated from) the vast majority of background values. As shown in Fig. 3f and g, both smoothing alone and CFE alone separate many signal fixels from the background; however the combination of smoothing and CFE achieves the best result (Fig. 3h).

### Computing a fixel analysis mask and obtaining correspondence across subjects

Whole-brain fixel–fixel connectivity is computed between all fixels in template space. We define the location and orientation of all template fixels using a “fixel analysis mask”. The approach used to generate this mask may differ depending on the diffusion model being analysed; however, ideally it should be representative of the population under investigation.

In this work (see Section 2.8), we compute the fixel analysis mask by first computing a population-specific FOD template using an iterative update approach (Raffelt et al., 2011). Accurate alignment of white matter is achieved by using FOD images to drive registration (Raffelt et al., 2011), and fixel orientations are corrected by reorientation of each FOD (Raffelt et al., 2012a). The FOD template is computed by averaging the spherical harmonic coefficients across all registered FOD images. To identify all fixels in the FOD template, we segment each FOD lobe using the method outlined in Smith et al. (2013). When correspondence and FOD alignment is poor across subjects (for example at the grey/white matter interface where inter-subject variation is greatest and registration is imperfect), the FOD lobes will not average constructively and their size will be small. We exclude these fixels from the analysis mask by thresholding the fixel AFD (as computed by integrating the FOD amplitude within each FOD lobe (Smith et al., 2013)). We note that thresholding fixels based on the AFD may undesirably exclude other fixels in crossing fibre regions that have low AFD (due to partial volume effects). We therefore compute the fixel analysis mask using a two-step process. First a relatively high AFD threshold (> 0.33) is used to exclude all unwanted fixels with poor correspondence near the grey matter interface. From this result we then compute a *3D voxel mask* defining all voxels that contain at least 1 fixel. The AFD threshold is then relaxed (> 0.1) to include fixels with small AFD values (i.e. those in crossing fibre regions) while excluding all fixels outside the 3D voxel mask.

The benefit of using a study-specific fixel analysis mask is that the location and orientation of fixels are representative of the population. The mask is therefore a good candidate for obtaining fixel correspondence across subjects by matching each template fixel to the nearest fixel in each of the subject images. Note that if no fixel exists in a subject image for a given template fixel (with a maximum angular tolerance of 30°), then it is assigned a quantitative value of zero. If a fixel exists in the subject that does not map to a template fixel then it is ignored.

### Statistical inference

In multiple testing problems, a family-wise error (FWE) refers to one or more false positives among the set of tests; such an error occurs if and only if the maximum over the set exceeds the decision threshold, implying that a suitable threshold to control the FWE rate (or equivalently, FWE-corrected p-values) can be obtained from the null distribution of the maximum-statistic (Nichols and Hayasaka, 2003). Permutation testing provides a non-parametric empirical null-distribution by recording the maximum-statistic computed for multiple permuted versions of the data, resting on the assumption of exchangeability under the null hypothesis (Holmes et al., 1996; Nichols and Holmes, 2002). For a general linear model, under the assumption that the (unobservable) errors are exchangeable, permutation of appropriate statistical residuals provides an approximate test that performs well in practice (Winkler et al., 2014). Here, complete images are permuted as a whole, preserving the complicated dependence structure, and the maximum is computed over all fixels' test statistics after CFE (i.e. the CFE procedure is applied to the statistic image for every permutation, effectively becoming part of the definition of the test statistic, as for TFCE or for the smoothed-variance t-map described by Nichols and Holmes, 2002). P-values are then assigned to each fixel by computing the proportion of the maximal CFE statistic distribution that is as-or-more extreme than the CFE value estimated using the original labelling of the data. For example, if 1000 permutations are performed (including the original labelling), and the original maximum is the 5th largest of these 1000, then its corrected p-value is 5/1000.

### Quantitative evaluation of CFE

We assessed the performance of CFE by generating a series of test statistic signals within an *in vivo* population-average template image. We explored the influence of CFE parameters *E, H* and *C* while varying the test statistic signal region-of-interest (ROI), signal-to-noise ratio (SNR) and smoothing spatial extent. Performance was assessed using a receiver-operator characteristic (ROC)-based evaluation.

#### *In vivo* data and pre-processing

Diffusion-weighted images were acquired from 80 healthy control subjects on a 3 T Siemens TIM Trio system (Erlangen, Germany), 60 diffusion directions, b = 3000 s/mm^2^, 2.3 mm. Motion correction, bias field correction and intensity normalisation were performed as described in Raffelt et al. (2012b). Fibre orientation distributions (FODs) were computed using robust constrained spherical deconvolution at l_max_ = 8 (Tournier et al., 2013).

#### Group-average FOD template and tractography

**Fig. 4:**
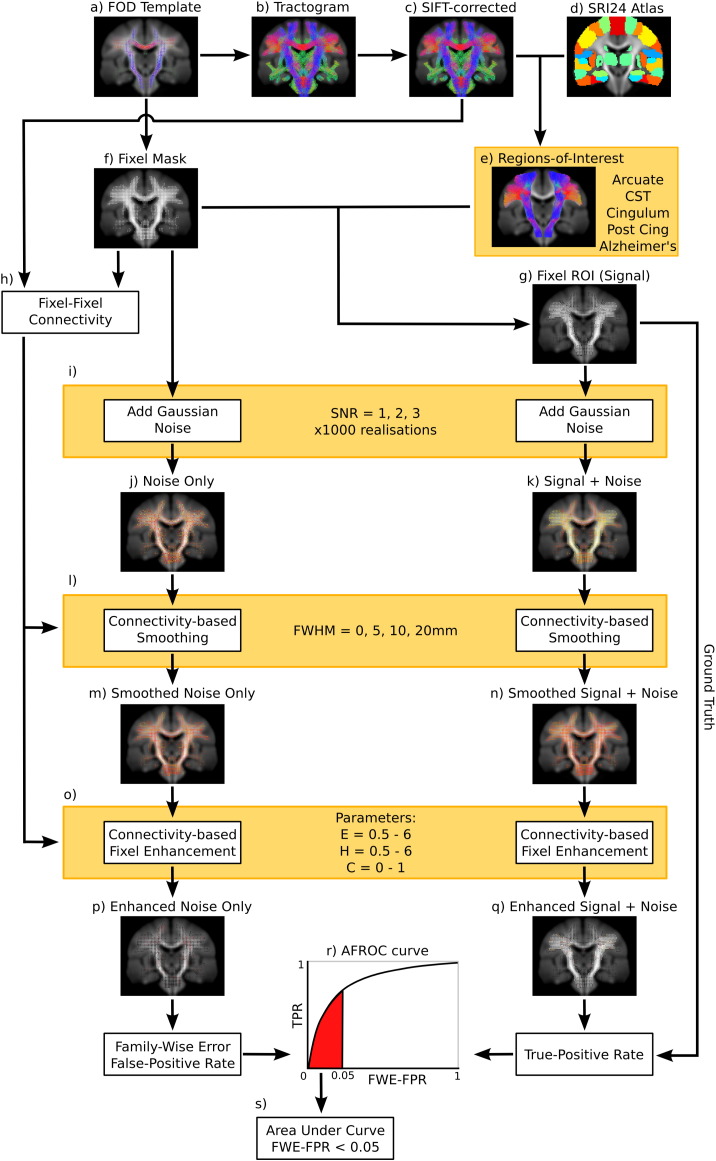
Schematic of the quantitative CFE evaluations performed using simulated test-statistic images. A FOD template (a) was used to generate a whole-brain tractogram (b) that was then filtered using SIFT (c). The SRI24 atlas (d) was used to extract tract ROIs (e), which were combined with a FOD template-derived white matter fixel-mask (f) to define fixel ROIs (g). h) For all mask fixels, connectivity to other fixels was computed using the tractogram in c. For each ROI, 1000 simulated test-statistic images were created by adding noise (i) to all fixels in f and g, to generate a noise only (j) and signal + noise image (k). Fixel images were smoothed with a range of kernel extents (l–n) and CFE enhanced (o) using a range of values for parameters *E*, *H* and *C*. Enhanced images (p and q) were evaluated by computing the area-under the curve (AUC) (s) of an AFROC curve (r).

All FOD images were registered to a group-average template using a FOD-based symmetric diffeomorphic registration algorithm (Raffelt et al., 2011) (Fig. 4a). During registration and the final spatial transformation, FODs were reoriented using apodised point spread functions (Raffelt et al., 2012a). Whole-brain probabilistic tractography was performed on the FOD template image to generate 30 million streamlines (Fig. 4b). This was performed using the iFOD2 tractography algorithm (Tournier et al., 2010), as part of the MRtrix software package (Tournier et al., 2012) (https://github.com/MRtrix3) (parameters: step size 0.625, angle 22.5, max length 250 mm, min length 10, power 0.5). To reduce tractography reconstruction biases we applied the spherical deconvolution informed filtering of tractograms (SIFT) method to give a final count of 3 million streamlines (Fig. 4c) (Smith et al., 2013).

#### Regions-of-interest

**Fig. 5:**
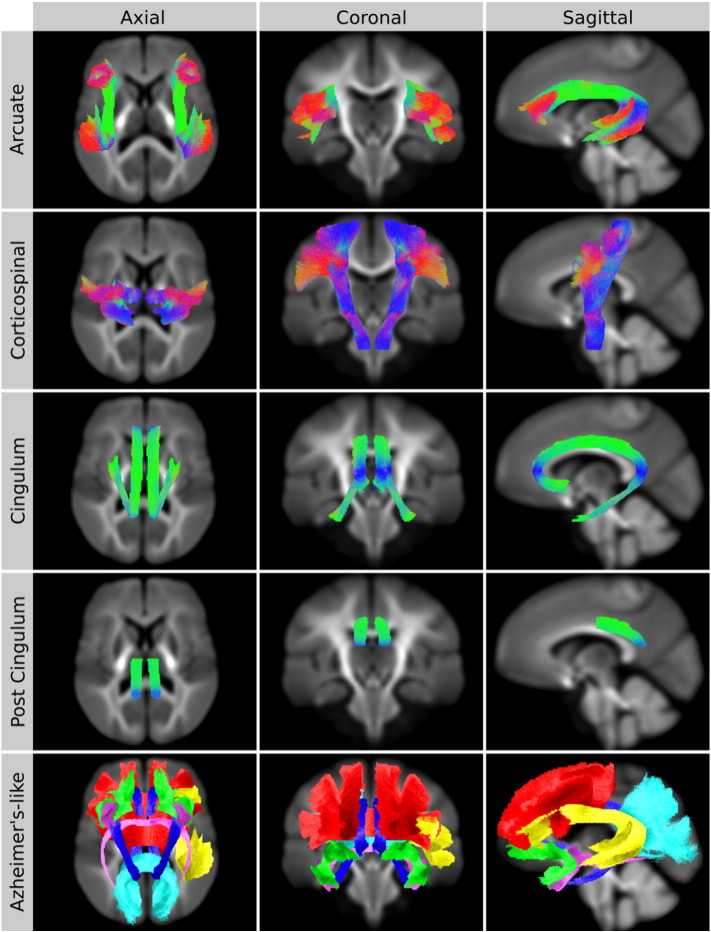
Fibre tractography regions-of-interest used to identify fixels with a test-statistic signal. All tracts were extracted from a whole-brain tractogram (Fig. 4c) using the SRI24 atlas (Fig. 4d) and edited by a neurologist. All tracts in rows 1–4 are coloured by streamline direction (red: left–right, blue: inferior–superior, green: anterior–posterior). The bottom row illustrates tracts that would be affected in an Alzheimer's-like pathology, chosen to simulate diseases that have a more global pathology. Alzheimer's-like fibre tracts include the left arcuate fasciculus (yellow), cingulum (dark blue), anterior commissure (pink), uncinate fasciculus (green), anterior corpus callosum (red), and posterior corpus callosum connecting the left and right precuneus (light blue).

We chose to evaluate CFE by generating a test-statistic signal in five different regions-of-interest (ROI) (Fig. 5). ROIs selected were the arcuate fasciculus, corticospinal tract, cingulum, posterior cingulum, and an Alzheimer's-like pathology. ROIs were selected to cover a broad range of properties (fibre bundle length, thickness, curvature and number of crossings). The arcuate fasciculus has a large proportion of crossing fibres with high posterior curvature (Fig. 5, top row). The corticospinal tract is a relatively large bundle that contains some crossings and a high degree of fanning (Fig. 5, 2nd row). The cingulum bundle is long and thin with a low proportion of crossing fibres (Fig. 5, middle row). The posterior cingulum was selected to test small and focal pathology (Fig. 5, 4th row). While it is our assumption that white matter pathology/maldevelopment generally should occur along the entire length of a bundle, the cingulum bundle contains many “on/off ramps” into the cingulate cortex, and therefore it is feasible that only a portion of the cingulum may be affected. The last ROI tested was an Alzheimer's-like pathology, chosen to represent diseases that affect several white matter bundles (Fig. 5, bottom row). Alzheimer's-like fibre bundles included the left arcuate fasciculus (yellow), cingulum (dark blue), anterior commissure (pink), uncinate fasciculus (green), anterior corpus callosum (red), and posterior corpus callosum connecting the left and right precuneus (light blue).

To identify each fibre ROI (Fig. 4e), streamlines were extracted from the template-generated tractogram using grey matter include-regions defined by the SRI24 atlas (Fig. 4d) (Rohlfing et al., 2010). The SRI24 atlas was co-registered to the group-average template using fractional anisotropy and mean diffusivity maps simultaneously (using the ANTS software package; http://picsl.upenn.edu/software/ants/). Spurious streamlines were removed with exclude-regions defined by a neurologist. In addition we cropped streamlines in regions where the streamline density was less than 2% of the maximum density within that tract. This ensured that final ROIs did not contain regions traversed by relatively few (probabilistically unlikely) streamlines.

#### Generating test-statistic images

We computed a white matter fixel mask as described in the section ‘Computing a fixel analysis mask and obtaining correspondence across subjects’ (Fig. 4f). A binary fixel signal image for each ROI (Fig. 4g) was created by mapping streamlines (Fig. 4e) to associated fixels in the template mask (Fig. 4f). We then generated 1000 instances of random Gaussian noise (N(0,1)) (Fig. 4i) to give 1000 ‘noise only’ (Fig. 4j) and 1000 ‘signal + noise’ images (Fig. 4k). Different SNR levels of the signal + noise images were created by modifying the signal in the ROI fixels (SNR = 1, 2, and 3).

#### Smoothing and enhancement parameters

To test the effect of the proposed connectivity-based smoothing, we smoothed the ‘noise only’ and ‘signal + noise’ images with kernels of 0, 5, 10, and 20 mm full width half maximum (FWHM) (Fig. 4l). The smoothed data (Fig. 4m, n) was renormalised so that the noise standard deviation was equal to 1. Variance renormalisation ensures that smoothing of the simulated test statistic image is equivalent to smoothing of the original data (as performed in a typical VBA) (Smith and Nichols, 2009). We tested CFE performance with various combinations of parameters *E*, *H* and C (Fig. 4o). Specifically, we selected *E* = 0.5, 1, 2, 3, 4, 5, 6, *H* = 0.5, 1, 2, 3, 4, 5, 6, and *C* = 0, 0.25, 0.5, 0.75, 1.0.

#### ROC-based evaluation

We assessed the performance of CFE using a receiver operator curve (ROC)-based approach. ROC curves are typically used to evaluate a *single inference* by plotting the true-positive rate (TPR; sensitivity) verses the false-positive rate (FPR; 1—specificity) while a discrimination threshold is varied. In fixel-based analysis, we are interested in the performance of *many inferences*, while controlling for the family-wise error rate. To account for these multiple comparisons we assessed CFE performance using the Alternative Free-response ROC (AFROC) method (Chakraborty and Winter, 1990) (as also performed in Smith and Nichols (2009)). The AFROC method controls the family-wise error rate (FWER) by defining the false-positive rate (FPR) as the fraction of realisations with *any* false positive fixels anywhere in the image, while the true-positive rate (TPR) is computed as the average number of true-positive fixels across realisations.

Specifically, the ROC curves (Fig. 4r) were computed by varying a threshold applied to the enhanced statistic images (Fig. 4p, q). The same thresholds were applied to the ‘enhanced noisy only’ image and ‘enhanced signal + noise’ image (between zero and the maximum enhanced signal + noise value).

To quantitatively assess each ROC curve we computed the area under the curve (AUC). As per Smith and Nichols (2009) we limit the AUC calculation to FPR values less than 0.05 (since we are not interested in FWER over 0.05) and divide the AUC by 0.05 so that it ranges from 0 to 1 (Fig. 4s). We visualised the AUC results using heat maps generated with the ggplot2 R software package (Core R Team, 2013)

### Application to motor neurone disease

To illustrate an *in vivo* application of the proposed CFE statistical inference method, we performed an AFD fixel-based analysis comparing a group of motor neurone disease (MND) patients with healthy controls. MND is characterised by progressive degeneration of motor neurons resulting in clinical symptoms that include muscular atrophy, muscular paralysis, and spasticity. Previous diffusion MRI studies have identified significant differences in white matter pathways involved in the motor system, including the corticospinal tract and corpus callosal fibres associated with the primary motor cortex. For a recent review of MND diffusion MRI studies see Foerster et al. (2012, 2013).

#### Participants, data and pre-processing

Participants included in this study were recruited as part of a MND study described in our previous work (Raffelt et al., 2012b). For a comprehensive description of participant details, acquisition protocols, and pre-processing methods the reader is referred to Raffelt et al. (2012b). However, for completeness we have included a brief summary of these details below.

We acquired data from 24 healthy control subjects and 24 patients with probable or definite MND, as defined by the revised El Escorial criteria (Brooks et al., 2000). All patients included in this analysis were classified as having upper motor neurone disease (Primary Lateral Sclerosis). Twenty-four healthy control participants were also recruited who had no history of hypertension or cerebrovascular disease and were not on any medications. All of the subjects gave their informed written consent, in line with the Declaration of Helsinki, and as approved by the local Human Research Ethics Committee.

MRI data were acquired using a 3 T Siemens Tim Trio (Siemens, Erlangen, Germany) and with a 12 channel head coil. The diffusion imaging parameters were: 60 axial slices, TR/TE 9200/112 ms, 2.5 mm slice thickness, 2.3 mm in plane image resolution, and an acceleration factor of 2. Sixty-four diffusion-weighted images (b = 3000 s/mm^2^), and one b = 0 image were acquired using echo planar imaging. Gradient encoding vectors were uniformly distributed in space using electrostatic repulsion (Jones et al., 1999). The acquisition time for the diffusion dataset was 9:40 min.

Pre-processing of diffusion MRIs included EPI correction (Jenkinson, 2003), motion correction (Raffelt et al., 2012c), bias field correction based on the b = 0 image (Tustison et al., 2010), and up-sampling by a factor of 2 using b-spline interpolation (Raffelt et al., 2012b). Diffusion MR images were intensity normalised across subjects based on the median b = 0 intensity within a white matter mask. Note that the corticospinal tract and mid body of the corpus callosum were manually excluded from the normalisation white matter mask since T2 hyper-intensities are observed in MND. FODs were computed using robust constrained spherical deconvolution at l_max_ = 8 (Tournier et al., 2013). As described in Raffelt et al. (2012b), we used a group average response function to estimate FODs in all subjects.

#### Fixel-based analysis

We compared AFD between the MND and control group over all white matter fixels. AFD is a quantitative measure derived from the FOD (Raffelt et al., 2012b). At typical diffusion gradient pulse durations (~ 30 ms) and high b-values (b = 3000 s/mm^2^), the FOD amplitude (i.e. the AFD) along a given direction is proportional to the intra-axonal volume of axons aligned with that direction. In this work we compute a fixel-specific measure of AFD by integrating the FOD within each lobe. As described in Smith et al. (2013), FOD lobes are first segmented based on FOD amplitude zero crossings, and the AFD of each lobe is integrated using a non-parametric numerical integration using a dense sampling of the FOD over a hemisphere.

Spatial normalisation of subjects and template-based tractography were performed as previously described in the section ‘Quantitative evaluation of CFE’. AFD data in each fixel were smoothed using the proposed connectivity-based smoothing (10 mm FWHM). The white matter analysis fixel mask and fixel correspondence was computed as previously explained in the section ‘Computing a fixel analysis mask and obtaining correspondence across subjects’.

To illustrate the effect of different CFE parameters on *in vivo* data, we performed several statistical tests. We chose a range of CFE parameters (*E* = 0.5, 2, 4, *H* = 0.5, 3, 6 and *C* = 0.5) based on the AUC results from the quantitative evaluations. Statistical inference was performed using a general linear model (GLM) and non-parametric permutation testing (Freedman and Lane, 1983; Nichols and Holmes, 2002; Winkler et al., 2014), with 5000 permutations. Significant fixels (FWE p < 0.05) were displayed using the *mrview* command in MRtrix 3 (https://github.com/MRtrix3).

#### Analysis using tract-based spatial statistics

Tract-based spatial statistics (TBSS) is currently the most commonly used method for VBA of white matter using diffusion MRI (Smith et al., 2006). Numerous clinical studies have used the tools available as part of the FSL software package to investigate population differences in tensor-derived indices. We therefore included an additional analysis to investigate the TBSS results using the MND cohort. All pre-processing steps were performed as described above. Fractional anisotropy images were computed with a non-linear tensor fit using MRtrix3 (https://github.com/MRtrix3). The default TBSS pipeline was used by performing registration, skeletonisation and statistical analysis as per the TBSS user guide (http://fsl.fmrib.ox.ac.uk/fsl/fslwiki/TBSS/UserGuide).

## Results

### Quantitative evaluation of CFE

**Fig. 6:**
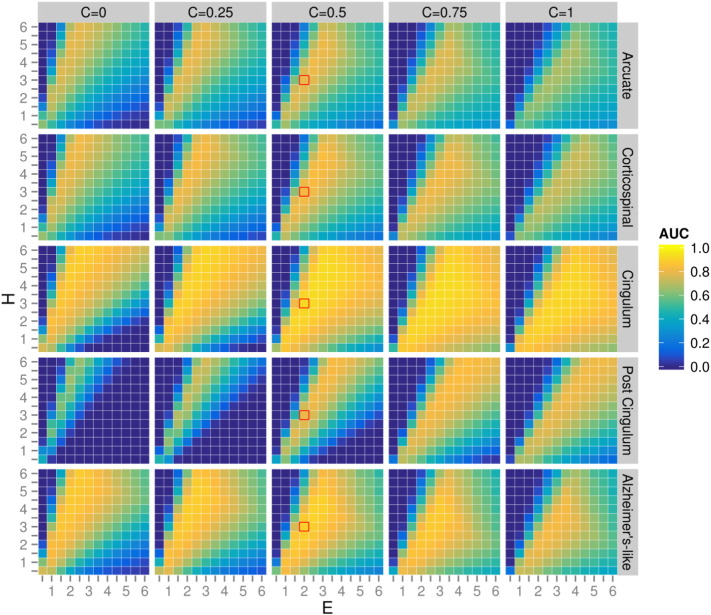
Influence of CFE parameters *C*, *E* and *H* on simulated pathology in five regions-of-interest. Plots are colour-coded by AFROC area under the curve (AUC). All plots were generated with SNR = 1 and a connectivity-based smoothing kernel of 10 mm FWHM. As indicated by the red squares, values *H* = 3, *E* = 2, *C* = 0.5 achieve good results for all regions-of-interest.

**Fig. 7:**
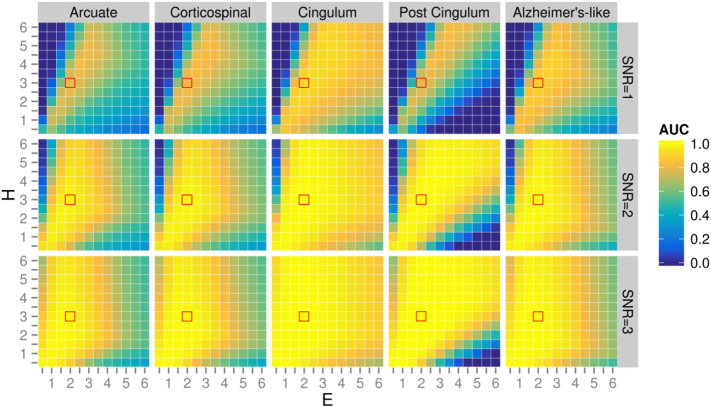
Influence of SNR and CFE parameters (*E* and *H*) on simulated pathology in five regions-of-interest. Plots are colour-coded by AFROC area under the curve (AUC). All plots were generated with *C* = 0.5 and a connectivity-based smoothing kernel of 10 mm FWHM. Red squares indicate recommended values (*H* = 3, *E* = 2).

**Fig. 8:**
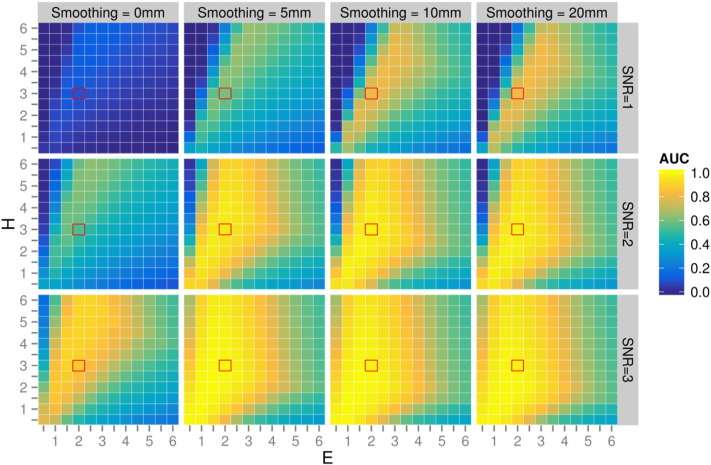
Influence of connectivity-based smoothing kernel size with different SNR and CFE parameters (*E* and *H*). Plots are colour-coded by AFROC area under the curve (AUC). All plots were generated by simulating a test-statistic signal in the arcuate fasciculus, and enhancing with *C* = 0.5. Red squares indicate recommended values (*H* = 3, *E* = 2).

We evaluated CFE performance with every combination of ROI, SNR, smoothing extent, and CFE parameters *C*, *E* and *H* shown by the orange boxes in Fig. 4. After careful investigation of all combinations, we included three figures to best illustrate the influence of each of the tested parameters (Figs. 6–8). For all Figs. 6–8 the heat map plots are coloured by the area under the curve (AUC) computed on the AFROC plots (FWE-FPR < 0.05).

Fig. 6 demonstrates the influence of CFE parameters *C*, *E* and *H* on a simulated test-statistic in 5 regions-of-interest (with a constant SNR = 1 and smoothing kernel = 10 mm FWHM). Fig. 7 demonstrates the influence of SNR on the optimal ratio of *E* verses *H* (with constant *C* = 0.5 and smoothing kernel = 10 mm). Fig. 8 demonstrates the influence of smoothing spatial extent with different effect sizes (with constant *C* = 0.5 and arcuate fasciculus ROI).

Based on these results a number of interesting observations were made:

1. Despite the fact that the ROIs have a broad range of properties (spatial extent, curvature and crossings), the optimal *H*, *E* and *C* are not heavily ROI-dependent (Fig. 6). As indicated by the red squares, values *H* = 3, *E* = 2, *C* = 0.5 achieves good results for all ROIs tested.
2. As *C* increases, the optimal ratio of *E* and *H* shifts towards a larger *E* (Fig. 6). This effect can be explained by the fact that a larger *C* value reduces the contribution of spatial extent to the enhancement relative to the height (by reducing the influence of long range fixels with lower connectivity).
3. The optimal *C* value is somewhat ROI dependent (Fig. 6). For example in the arcuate, corticospinal and Alzheimer's like ROIs, higher *C* values have reduced AUC values. However in both cingulum bundle ROIs lower *C* values perform poorly.
4. At a higher SNR, better AUC values are obtained over a wider range of *E* and *H* values (Fig. 7). Values *H* = 3, *E* = 2 give good results for all SNRs (as indicated by the red squares).
5. As shown in Fig. 8, connectivity-based smoothing improves the AUC results, but only up to a smoothing extent of 10 mm FWHM. There is no change in AUC when increasing the smoothing extent from 10 to 20 mm. Fig. 8 demonstrates the influence of smoothing only on the arcuate fasciculus ROI; however this trend was observed for all ROIs tested (data not shown).

### Motor neurone disease results

#### Fixel-based analysis results

**Fig. 9:**
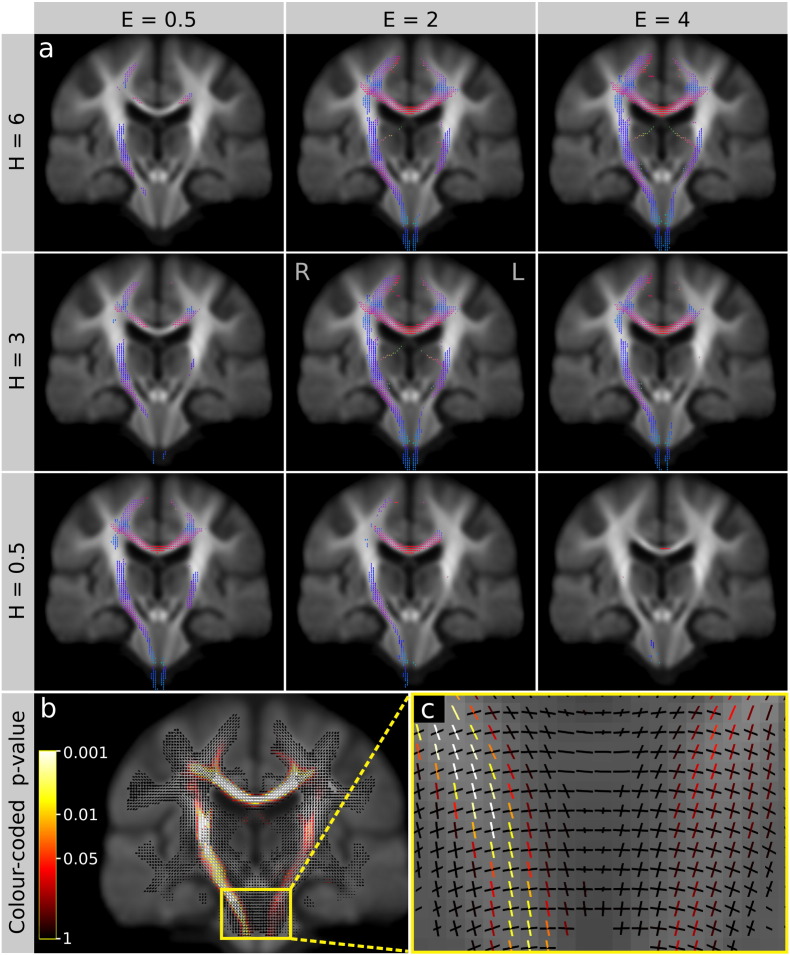
Fixel-based analysis results demonstrating a significant decrease (FWE-corrected p < 0.05) in apparent fibre density (AFD) in motor neurone disease (MND) patients compared to healthy controls. a) Significant fixels detected using various combinations of CFE parameters E and H. Fixels are coloured by direction, red: left–right, blue: inferior–superior, green: anterior–posterior. b) Fixels coloured by FWE-corrected p-value. c) Zoomed in region of the pons. As shown by the many crossing fibres in this region, the proposed CFE-based method enables fibre tract-specific analysis by attributing p-values to each fixel in voxels containing multiple fibre populations.

As shown in Fig. 9, a significant decrease in AFD was observed in motor neurone disease patients compared to healthy controls. All significant fixels in the brain were projected onto a coronal slice, coloured by fixel orientation (red: left–right, blue: inferior–superior, green: anterior–posterior) and overlaid on a single coronal slice of the mean AFD template image.

As expected the affected fixels were restricted to the motor pathways, namely the corticospinal tract and the interhemispheric callosal fibres interconnecting the left and right motor cortex. In addition we observed a significant reduction in AFD in the fornix. This is an interesting finding since many studies have linked MND with frontotemporal dementia, a disease that affects episodic memory and the fornix (Hornberger et al., 2012).

As demonstrated by the spatial extent of the significant region, the sensitivity of different CFE parameter combinations matches the trend observed in the simulation results shown in Fig. 6. We note that CFE values of *H* = 3, *E* = 2 and *C* = 0.5 (those that give consistently good results in the simulations) result in a large spatial extent, including many fornix fixels.

Fig. 9b illustrates a single slice of fixels colour-coded by p-value. As shown by the zoomed in region of the pons (Fig. 9c), the CFE method detects a group difference in fixels specific to the corticospinal tract, while the transpontine fibres are not statistically significant.

#### Tract-based spatial statistic results

**Fig. 10:**
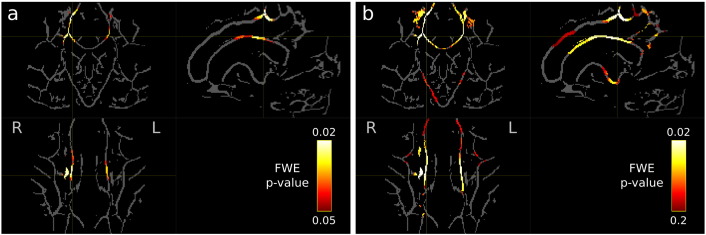
Tract-based spatial statistic (TBSS) results demonstrating a reduction in fractional anisotropy in the MND population compared to controls. a) Significant voxels (p < 0.05) are overlaid on the template FA skeleton. Differences were observed in the corpus callosum region associated with the motor cortex, with no voxels being significant in the corticospinal tract. b) When the p-value threshold is relaxed to 0.2, supra-threshold voxels are observed in the corticospinal tract; however at this threshold other regions not typically associated with MND are also supra-threshold. For both images the orientation shown is coronal (top left), sagittal (top right) and axial (bottom left).

Shown in Fig. 10 are the results from the TBSS analysis on the MND cohort. A significant decrease (p < 0.05) was detected in FA in MND patients compared to controls in the corpus callosum motor pathways (Fig. 10a). No significant differences were detected in the corticospinal tract. Supra-threshold voxels could be observed in the corticospinal tract by relaxing the p-value threshold to p < 0.2 (Fig. 10b); however, as might be expected at such a lenient threshold, many voxels are also then supra-threshold in regions that are not typically associated with MND.

## Discussion

We have outlined a novel connectivity-based fixel enhancement method for multi-subject whole-brain analysis of quantitative measures derived from higher-order diffusion MRI models. The CFE approach uses tractography-derived information to smooth and enhance between fixels that are *structurally connected* (and therefore likely share underlying anatomy and pathology). This is in contrast to 3D cluster-based methods (including TFCE), where a voxel may contribute to the enhancement of another if they are *spatially connected* by coexisting within a supra-threshold cluster (even if both voxels belong to different fibre tracts). In addition to the bundle-specific smoothing and enhancement, the primary motivation behind the CFE method is the ability to perform tract-specific statistical inference at an individual fixel level.

### Quantitative evaluation of CFE

Using *in vivo* data with a simulated test-statistic signal, we have demonstrated that the optimal CFE parameters are relatively insensitive to the signal ROI and SNR (Fig. 7). This is encouraging for future fixel-based analyses since close to maximum sensitivity should be obtained for most studies with *H* = 3, *E* = 2 and *C* = 0.5.

In all simulations larger AUC values were obtained with *E* > 1, which causes the enhancement to increase more than linearly with extent size (Figs. 6–8). This is in contrast to the original TFCE method (Smith and Nichols, 2009), where the recommended *E* = 0.5 causes enhancement to be scaled less than linearly with extent size. In 3D VBA of grey matter *E* < 1 is desirable because *“at the lowest values of h the sections* (clusters) *can become very large, but these large low areas of support are not providing very useful spatial specificity”* (Smith and Nichols, 2009). However, in CFE the extent is constrained to anatomically related fixels by tractography-based connectivity. Therefore at low values of *h*, unrelated fibre bundles cannot enhance each other.

Because we weight each fixel's contribution to the enhancement based on the probabilistic streamline connectivity, *c_fi_* (Eq. (3)), fixels with larger connectivity values (i.e. those that we are more certain share underlying anatomy) contribute more to the enhancement. In addition we raise *c_fi_* to the power of *C* to tune the influence of connectivity; for example when *C* < 1 the contribution from lower connectivities (e.g. over long ranges) is increased. As shown by Fig. 6, *C* = 0.5 gives good results for all ROIs. When *C* > 0.5 the arcuate, corticospinal, and Alzheimer's-like ROIs have a reduced AUC, while the two cingulum ROIs have a reduced AUC when *C* < 0.5. A possible explanation for the different behaviour observed in the cingulum ROIs is the mismatch between our simulated signal and the cingulum tractography. The signal was simulated in only the ‘core’ of the cingulum bundle (Fig. 5), however the tractography streamlines branch frequently along the entire length into the cingulate cortex (as they do in reality), which results in many weakly connected fixels located outside the ROI. A larger *C* value still enables strongly-connected core fixels to contribute to the enhancement, while decreasing the contribution of more weakly connected fixels.

As shown by the simulation results in Fig. 8, connectivity-based smoothing improved AUC values up to a smoothing kernel of 10 mm FWHM. We note that a FWHM = 10 mm kernel is relatively large compared to the sigma = 1.5 mm (FWHM = 3.5 mm) suggested in TFCE (Smith and Nichols, 2009), however connectivity-based smoothing ensures minimal blurring occurs across unrelated fibre tracts, as discussed earlier in relation to the E parameter.

The simulations included an Alzheimer's-like ROI to investigate CFE performance with a widespread pathology containing several fibre bundles. As shown in Figs. 6 and 7, the Alzheimer's-like ROI gives a similar relationship between *E* and *H* to the other ROIs tested. This is in contrast to what would be expected in TFCE, where an extensive pathology is likely to benefit from a larger *E/H* ratio. The relative insensitivity of CFE input parameters to small vs. extensive multi-bundle pathology is likely another consequence of the tract-specific enhancement.

### Motor neurone disease

The proposed CFE method has been recently applied in preliminary analyses of Alzheimer's disease (Raffelt et al., 2013), temporal lobe epilepsy (Raffelt et al., 2014c), adolescents born preterm (Raffelt et al., 2014a), grey matter heterotopia (Farquharson et al., 2014), Dravet syndrome (Raffelt et al., 2014b), and glaucoma (Raffelt et al., 2015), however this is the first time we have used CFE to study MND (Fig. 9). The significant reduction in AFD of the corticospinal tract of MND patients corroborates histopathological findings (Hughes, 1982), and clearly demonstrates the tract specificity of the CFE method in the brain stem region (Fig. 9c). The MND results in Fig. 9a support the CFE simulations with significant group differences being widespread with *H* = 3, *E* = 2, and *C* = 0.5. We note that in these results the group effect is more extensive in the right corticospinal tract (left side of the image) compared to the left corticospinal tract. This is an encouraging finding given that most patients in this MND cohort reported a left-sided onset of disease (15 left, 5 bilateral, 9 right).

### Inter-dependence of structurally connected fixels

The CFE method assumes that group effects in white matter should be correlated along a fibre bundle since the underlying axons should experience similar pathology along their entire length. While this assumption is sound for most developmental and neurodegenerative diseases, it may not hold for focal lesions found in diseases such as multiple sclerosis, stroke or traumatic brain injury. However, we point out that these diseases tend to be less suited for all VBA methods due to the spatial heterogeneity of the lesions across subjects. We also note that axon degeneration secondary to the site of the lesion is often of more interest, which is likely to be correlated along a fibre bundle's length.

By not requiring that fixels are spatially connected by supra-threshold fixels, a benefit of the CFE method is that two distant regions of the same fibre bundle may enhance each other even if the interconnecting fixels are sub-threshold. While this is unlikely to occur if axons are affected along their entire length, it is feasible that non-stationary variance (for example at fibre crossings) may reduce the test-statistic of interconnecting fixels that would otherwise prevent the co-enhancement of distant yet structurally related regions.

### Fixel analysis mask construction and fixel correspondence

When performing a traditional 3D voxel-based analysis, a mask is often used to restrict the analysis to voxels of interest. In the case of a fixel-based analysis, not only do we need to identify the voxel locations to be investigated, but also the number and orientation of fixels within the voxels.

In this work we compute the fixel analysis mask by segmenting the study-specific FOD template, then thresholding the fixel AFD. Previous work suggests that using an unbiased study-specific template may give better sensitivity and specificity to detect white matter abnormalities (Van Hecke et al., 2011). An additional benefit in the case of fixel-based analysis is that the fixel orientations computed from the FOD template will also be representative (an average) of the population. We note that even with a robust neighbourhood-based FOD estimation (Tournier et al., 2013), combined with FOD registration and reorientation (Raffelt et al., 2011, 2012a), fixel orientations may still vary across subjects. Correspondence is therefore achieved by matching the study-specific template fixel orientation to the closest fixel in all subjects (using a maximum angular tolerance of 30 degrees). This can be thought of as a projection step in the angular domain (akin to a TBSS spatial projection).

Generation of the fixel mask uses a two-step approach to empirically select AFD thresholds to ensure that fixels at the grey/white interface (where the AFD and fixel orientation across subjects is variable due to imperfect registration and partial volume with grey matter) are excluded, while fixels within crossing fibre regions in deep white matter are included. It is worth emphasising that the issue of choosing the optimal analysis mask threshold is not unique to the proposed fixel-based analysis method (Ridgway et al., 2009). However the advantage of white matter fixel-based analysis (e.g. compared to grey matter voxel-based analysis) is that white matter axons are extended in nature, and therefore while false-negative fixels may arise from increased variance at the periphery or an inappropriate mask threshold, group differences are still likely to be detected in more central (connected) regions where good registration and fixel correspondence is obtained.

### Analysis of quantitative measures other than AFD

While we have investigated fixel-specific AFD differences in this study, the proposed CFE method can be applied to other fixel-specific quantitative measures derived from the ‘composite hindered and restricted model of diffusion’ (CHARMED) model (Assaf and Basser, 2005) and the cube and sphere multi-fascicle model (CUSP-MFM) (Scherrer and Warfield, 2012). In other preliminary work (Raffelt et al., 2014c), we used the proposed CFE method to investigate population differences in white matter morphometry, using in a novel technique called fixel-based morphometry (FBM). In FBM, the fixel-specific measure is based on morphometric differences in fibre-bundle cross-sectional area derived entirely from the non-linear transformations of each subject to the template.

We also note that while the proposed method was designed to investigate fixel-specific measures, the CFE method could also be used to investigate voxel-average quantities (e.g. myelin water fractions). This would be achieved by mapping the value at each voxel to all fixels within that voxel, then using CFE as described in this work. While this is not optimal since the quantitative measure is not fixel-specific in regions with crossing fibres, smoothing and enhancement would still be more tract-specific than if performed using traditional 3D smoothing and clustering, and the estimated p-value of fixels within the same voxel will differ based on the different connectivity-derived neighbourhoods.

### Contrast to previous methods

The reduced sensitivity in the TBSS result (Fig. 10 vs. Fig. 9) may be due to differences in the quantitative measure (FA vs AFD), the use of a fixel vs. voxel-based measure, sub-optimal tensor b-value (3000 s/mm^2^ in this study), the registration algorithm, tract-specific vs isotropic smoothing, or the statistical method (projection vs whole-brain, spatial vs connectivity enhancement). To eliminate most of these confounds and compare the smoothing and statistical method only, one could compare CFE to the multi-fibre version of TBSS approach (Jbabdi et al., 2010). This would require images to be aligned with the FOD-registration-derived warps, before projecting fixel-specific AFD values onto the FA-skeleton. However, the multi-fibre TBSS method can only analyse a maximum of two fixels per voxel. The centrum semiovale (a region containing significant fixels in the MND result) contains many voxels with three fixels (corticospinal tract, superior longitudinal fasciculus, and corpus callosum) and therefore it is not possible to correctly convert/map AFD fixel images to the MF-TBSS two-fixel format. In addition to this issue, a comprehensive and fair comparison of TBSS and CFE sensitivity and specificity should ideally be performed using ground truth simulated pathology (in several white matter regions) and is therefore beyond the scope of this current work.

We included an ‘off-the-shelf’ TBSS analysis in this paper since TBSS is the most commonly used method for voxel-based analysis in diffusion MRI. However, TBSS is very different in kind to the whole-brain fixel-based analysis presented in this work. TBSS is often cited as a whole-brain voxel-based method; however only a very small percentage of the white matter is investigated since the skeletonisation and projection step is based on regions with high FA (and therefore the majority of white matter voxels with crossing fibres and low FA are excluded from analysis). Part of the motivation behind the projection step in TBSS is to improve alignments in FA-based registration. However, recent work suggests that using more advanced DTI registration improves white matter alignment, while the TBSS projection step actually reduces detection accuracy and produces less biologically plausible results *in vivo* compared to a whole-brain VBA (Bach et al., 2014; De Groot et al., 2013; Schwarz et al., 2014). In this present work, we perform registration using FOD images that contain high contrast within white matter (Raffelt et al., 2011), and therefore good correspondence is achieved across subjects at the fixel-level for all major fibre tracts. Furthermore, the TBSS projection step may also fail to capture group differences in pathology (where the FA is low), since the projection step is based on high FA voxels. In the context of the multi-fibre TBSS method (Jbabdi et al., 2010), high FA voxels are more likely to contain single fibre populations, and therefore voxels with multiple fixels (that typically have low FA) are also less likely to be included in the analysis.

Tract-based spatial statistics (TBSS) is the most commonly used method for VBA of white matter using diffusion MRI (Smith et al., 2006). Furthermore, TBSS is insensitive to cases where pathology may affect a low FA subset of axons belonging to a bundle (since the projection step preferentially selects high FA voxels). The fixel-based analysis method proposed in this work tests all white matter regions and therefore does not suffer from this limitation.

More recent work has extended the TBSS framework to investigate higher order models with multiple fixel-specific quantitative measures per voxel (Jbabdi et al., 2010). However this approach still relies on tract skeleton projection and therefore only tests a relatively small fraction of white matter. Moreover, the tract skeletonisation and projection procedure rely on high FA voxels that are more likely to contain single fibre populations, and therefore voxels with multiple fixels are less likely to be included in the analysis.

To our knowledge, the only other VBA method to test fibre-specific information from higher order diffusion MRI models was presented in our previous work (Raffelt et al., 2012b). This approach enabled group comparisons of AFD that was derived by sampling the FOD uniformly over many directions within each voxel. That method is therefore limited to quantitative measures derived from continuous spherical functions, and is very sensitive to subtle miss alignments in fibre orientations due to imperfect image registration. In contrast, the proposed CFE method is not sensitive to small fibre orientation misalignments since we obtain fixel correspondence using the group-average fixel analysis mask with an angular tolerance of 30°. Furthermore, since a single scalar quantity is tested per fixel, other fixel-specific diffusion MRI measures can be investigated using CFE (as discussed in Section 4.5).

### Implementation considerations

We implemented the proposed CFE statistical inference method in a command called *fixelcfestats*, as part of the freely-available open-source cross-platform MRtrix software package (https://github.com/MRtrix3). The *fixelcfestats* command is multi-threaded and therefore computation time decreases linearly with the number of CPU cores. At typical DWI resolution (2.5 mm), 5000 permutations can be completed in several hours on a standard desktop PC. On high resolution data (1.25 mm) 5000 permutations can be completed overnight, however more memory is required (> 64 GB).

When computing the whole-brain tractogram the total number of streamlines should be sufficient to achieve precise fixel–fixel connectivity estimates. In this work we used 30 million streamlines, which were subsequently filtered with SIFT to a total 3 million streamlines (Smith et al., 2013). We chose 3 million since this was the maximum possible given memory limitations, however in practise 2 million streamlines should be sufficient. Note that SIFT is an important step to remove tractography biases (e.g. over seeding in large tracts) and improve fixel–fixel connectivity estimates. Other work conceptually related to SIFT suggests that weighting streamlines to fit the underlying data may also help to remove false-positive connections (Daducci et al., 2014).

### Limitations

In a similar approach to Smith and Nichols (2009), we assessed CFE performance using a test-statistic image generated by adding *stationary* Gaussian noise to a fake signal, and smoothing with a stationary kernel. However *in vivo* generated test-statistic images are inherently *nonstationary* due to spatial variations in scanner SNR and anatomical variability. Nonstationary smoothness is problematic because larger clusters are expected in smoother areas by chance. Stationary random field theory cluster-based approaches can fail to control the FWER in such cases. The random field theory approach has been adapted for non-stationary smoothness (Worsley et al., 1999), but has been found to work well only for high degrees of freedom and high smoothness (Hayasaka et al., 2004). Importantly, permutation-based approaches control FWER in all cases, though versions wrongly assuming stationarity will exhibit non-stationary sensitivity, motivating non-stationary versions. Hayasaka et al. (2004) evaluated a permutation approach that used the estimated local smoothness, while Salimi-Khorshidi et al. (2011) adjust cluster sizes using a resampling-based estimate of nonstationarity. The latter approach can be employed to adjust both cluster sizes and TFCE (or CFE) output. It is important to note that while cluster-extent is strongly affected by non-stationary smoothness, methods that combine extent and height, such as cluster-mass and TFCE, are expected to be more robust to non-stationary smoothness, since the larger clusters in smoother areas will tend to have reduced heights. This was supported by experimental results for TFCE by Salimi-Khorshidi et al. (2011), and is likely to hold for CFE as well. Nevertheless, the lack of non-stationary effects in our simulations must be acknowledged as a limitation, and we plan to investigate approaches like that of Salimi-Khorshidi et al. (2011) in future work. Our results on real (presumably non-stationary) MND data provide reassurance that the optimal parameters identified in the simulations should still be appropriate for *in vivo* studies.

Edden and Jones (2011) identified an orientational bias in the statistical sensitivity of TBSS method: fibre pathways in the tract-skeleton that are oblique to the image grid are represented with more voxels and are more likely to be significant than those aligned with the grid. In CFE there is no tract skeleton, but when computing the fixel–fixel connectivity, more streamlines are likely to traverse through a voxel when the fibre is oriented obliquely. It's therefore possible that an oblique fixel may be structurally connected to more fixels and have a larger extent, *e*, compared to a fixel aligned with the image axis. Future experiments will investigate this, alongside potential adjustments like that of Salimi-Khorshidi et al. (2011).

Our simulations encompassed 50,700 combinations of ROI, effect size, smoothing and CFE parameters. However, we did not explore the effect of different tractography algorithms and parameters on the computed fixel–fixel connectivity matrix. Certain parameters may influence the tractography output (e.g. probabilistic spread) and therefore the accuracy and sparsity of the connectivity matrix. Further investigation on this is warranted but is beyond the scope of this work.

## Conclusion

In this work we have introduced a novel approach for whole-brain statistical analysis of fixel-based measures derived from higher order diffusion MRI models. The CFE method exploits connectivity information derived from probabilistic tractography to ensure pre-smoothing and enhancement is performed using fixels that are likely to share underlying anatomy and pathology. Simulations suggest that enhancement parameters are relatively insensitive to the simulated pathology ROI and SNR, and therefore we can recommend a single set of parameters (*H* = 3, *E* = 2, *C* = 0.5) that should give near optimal results in future studies where the group effect is unknown. We demonstrated the proposed method by comparing a group of MND patients to control subjects and achieve good results with the simulation-derived parameters. In addition to providing tract-specific smoothing and enhancement, the key benefit of the CFE method is to permit fixel-specific statistical inference that should yield more interpretable results in white matter regions that contain crossing fibres.

## Footnotes

1 Note that this may not be true for lesions in diseases such as Stroke and Multiple Sclerosis. However, these lesions tend to be spatially heterogeneous and therefore less suited to multi-subject VBA.

2 Previous publications have used the word ‘fibre’ (Assaf and Basser, 2005; Behrens et al., 2007)(Assaf and Basser, 2005), ‘fascicle’ (Scherrer and Warfield, 2012) or ‘fibre population’ (Behrens et al., 2007; Raffelt et al., 2012b) to refer to a specific population of fibres within a single voxel. However, these terms can be ambiguous in certain contexts. For example, when attributing a quantitative measure to a ‘fibre’, it may be misinterpreted as belonging to the entire fibre bundle. Here, we introduce a new word ‘fixel’ to eliminate this ambiguity when discussing fixel-specific measures and fixel-based analysis (FBA).
